# Supplementary material for: Mixed-methods assessment of engagement with a digital intervention: The Wrapped feasibility randomised controlled trial
Source: PLOS Digit Health. 2026 Feb 12;5(2):e0001202. doi: 10.1371/journal.pdig.0001202 (PMC12900356; doi:10.1371/journal.pdig.0001202)
Supplement: S1 File — (DOCX) [file pdig.0001202.s001.docx]

### S1 File. Expressed Need and Component Assignment

Barriers to condom use were measured in both the baseline survey and at website registration via the tailoring items (see below for more detail on the items asked). Intervention components were assigned based on expressed needs represented via the tailoring items, not the baseline survey. In order to explore if the tailoring items completed at website registration were reflective of responses at baseline, baseline survey responses were examined and categorised to identify participants who answered “Disagree” or “Strongly disagree” to items that matched the expressed need of a tailoring item.

Table A demonstrates how each component would have been assigned via the tailoring items or by the survey items. A cross tabulation was performed to identify how many participants were allocated each intervention component based on identified need expressed in the tailoring items as compared with identified need expressed in the baseline survey.

Table A. Assignment of Intervention Components by Expressed Need via Tailoring Items and Survey Items

| **Tailoring Items** | **Survey Items*** | **Ages** | **Components** |
| --- | --- | --- | --- |
| I can’t always get the type of condoms I want | I am confident that.. I can get hold of more condoms when I need them | All ages | Condom Ordering Service |
| I find condoms expensive to buy | The cost of condoms has not been an issue for me | All ages | Condom Ordering Service |
| I find buying condoms embarrassing | I am confident that.. I can get hold of more condoms when I need them | All ages | Condom Ordering Service |
| I don’t always have a condom on me when its needed | I have kept condoms in places where I'm most likely to have sex (e.g. bedroom)  I have always had condoms on me when I'm out and about | All ages | Condom Carrier |
| I find it awkward or difficult letting someone know that I want to use condoms | I am confident that.. I can discuss condom use with any partner I might have | All ages | Discussing Condoms Videos |
| I’m not always able to put a condom on with confidence and ease | I am confident that.. I can correctly put a condom on myself or a partner | All ages | Condom Demo Video |
| I find using condoms a turn-off |  | Under 18 | - |
|  |  | 18 and over | Real Life Videos |
| I find using condoms interrupts the flow of sex | Using condoms will interrupt the flow of sex | Under 18 | Condom Demo Video; Discussing Condoms Videos |
|  |  | 18 and over | Condom Demo Video; Discussing Condoms Videos; Real Life Videos |
| Condoms make sex less enjoyable or pleasurable for me | Sex with condoms can be pleasurable for me  Sex with condoms can be enjoyable | Under 18 | Condom Demo Video; |
|  |  | 18 and over | Condom Demo Video; Real Life Videos |
| Condoms make sex less enjoyable or pleasurable for the person I’m with | Sex with condoms can be pleasurable for my partner | Under 18 | Condom Demo Video |
|  |  | 18 and over | Condom Demo Video; Real Life Videos |

*Participant would be assigned the component if they responded Disagree or Strongly Disagree

### Tailoring Items

1. I can’t always get the type of condoms I want (Yes/No)
2. I find condoms expensive to buy (Yes/No)
3. I find buying condoms embarrassing (Yes/No)
4. I don’t always have a condom on me when its needed (Yes/No)
5. I find it awkward or difficult letting someone know that I want to use condoms  (Yes/No)
6. I’m not always able to put a condom on with confidence and ease (Yes/No)
7. I find using condoms a turn-off (Yes/No)
8. I find using condoms interrupts the flow of sex  (Yes/No)
9. Condoms make sex less enjoyable or pleasurable for me (Yes/No)
10. Condoms make sex less enjoyable or pleasurable for the person I’m with (Yes/No)

### Baseline Survey Items Regarding Barriers to Using Condoms

1. I can get hold of more condoms when I need them (Strongly agree/Agree/Neither agree nor disagree/Disagree/Strongly disagree)
2. The cost of condoms has not been an issue for me (Strongly agree/Agree/Neither agree nor disagree/Disagree/Strongly disagree)
3. I have kept condoms in places where I’m most likely to have sex (e.g. bedroom) (Strongly agree/Agree/Neither agree nor disagree/Disagree/Strongly disagree)
4. I have always had condoms on me when I'm out and about (Strongly agree/Agree/Neither agree nor disagree/Disagree/Strongly disagree)
5. I can correctly put a condom on myself or a partner (Strongly agree/Agree/Neither agree nor disagree/Disagree/Strongly disagree)
6. Using condoms will interrupt the flow of sex (Strongly agree/Agree/Neither agree nor disagree/Disagree/Strongly disagree)
7. Sex with condoms can be pleasurable for me (Strongly agree/Agree/Neither agree nor disagree/Disagree/Strongly disagree)
8. Sex with condoms can be pleasurable for my partner (Strongly agree/Agree/Neither agree nor disagree/Disagree/Strongly disagree)
9. Sex with condoms can be enjoyable (Strongly agree/Agree/Neither agree nor disagree/Disagree/Strongly disagree)
10. I can discuss condom use with any partner I might have (Strongly agree/Agree/Neither agree nor disagree/Disagree/Strongly disagree)
