# Supplementary material for: Mixed-methods assessment of engagement with a digital intervention: The Wrapped feasibility randomised controlled trial
Source: PLOS Digit Health. 2026 Feb 12;5(2):e0001202. doi: 10.1371/journal.pdig.0001202 (PMC12900356; doi:10.1371/journal.pdig.0001202)
Supplement: S2 File — (DOCX) [file pdig.0001202.s002.docx]

### S2 File. Months 3 and 12 Survey Items

### Month 3 Survey Items Regarding Website

1. After completing the first survey, we emailed you a link to a website about condom use called 'Wrapped'. Did you visit this? (Yes/No)

If Yes:

1. How useful did you find this website? (Not at all useful/Slightly useful/Moderately useful/Very useful/Extremely useful)
2. How would you rate the design of this website? (Very poor/Poor/Average/Good/Very good)

### Month 12 Survey Items Regarding Website

Did you receive any of the following items via the Wrapped website*?
Please select all that apply (Sample box of condoms/Monthly delivery of condoms and lube/Condom carrier)

*Please note: different people were given access to different items from the Wrapped website depending on their needs. You therefore may not have had the chance to order all of these items.

If Sample Box Selected:

1. Did you try any of the condoms? (Yes/No)
2. Did you try any of the lube? (Yes/No)
3. Did you look at the leaflet that came inside the box? (Yes/No)
4. Have you used the box for storing condoms? (Yes/No)

If Month delivery of condoms and lube Selected:

1. How many times did you place an order? (Once/More than once)
2. How much of the condoms/lube have you used? (I used all of it/I used most of it/I used some of it/I haven't used any of it)
3. How much of the condoms/lube in your most recent order have you used? (I used all of it/I used most of it/I used some of it/I haven't used any of it)

If Condom Carrier Selected:

1. Have you used the carrier? (Yes, to carry condoms/Yes, but not to carry condoms/No, I haven't used it)
2. Is the carrier attached to your keys/bag? (Yes/No)
3. Why is this? (select all that apply) (I didn't like the colour/I didn't like the design/Not discreet enough/Other reason)

If Other Reason Selected:

1. Please tell us why (Open text box)
