# Supplementary material for: Mixed-methods assessment of engagement with a digital intervention: The Wrapped feasibility randomised controlled trial
Source: PLOS Digit Health. 2026 Feb 12;5(2):e0001202. doi: 10.1371/journal.pdig.0001202 (PMC12900356; doi:10.1371/journal.pdig.0001202)
Supplement: S3 File — (DOCX) [file pdig.0001202.s003.docx]

Fig A. Wrapped website access flowchart

Fig B. Sample Pack orders flowchart

Fig C. Condom Ordering Service flowchart

Fig D. Condom Carrier orders flowchart

*1 participant watched the video in full twice (on two separate visits)

Fig E. Condom Demo Video flowchart

Fig F. Discussing Condoms Videos flowchart

Fig G. Real Life Videos flowchart
